# Control of patterns of symmetric cell division in the epidermal and cortical tissues of the *Arabidopsis* root

**DOI:** 10.1242/dev.129502

**Published:** 2016-03-15

**Authors:** Yanwen Zhang, Michail Iakovidis, Silvia Costa

**Affiliations:** Department of Cell and Developmental Biology, John Innes Centre, Norwich NR4 7UH, UK

**Keywords:** Tissue patterning, *TON1a*, Meristem, Epidermis, Cortex, Preprophase band

## Abstract

Controlled cell division is central to the growth and development of all multicellular organisms. Within the proliferating zone of the *Arabidopsis* root, regular symmetric divisions give rise to patterns of parallel files of cells, the genetic basis of which remains unclear. We found that genotypes impaired in the *TONNEAU1a* (*TON1a*) gene display misoriented symmetric divisions in the epidermis and have no division defects in the underlying cortical tissue. The *TON1a* gene encodes a microtubule-associated protein. We show that in the *ton1a* mutant, epidermal and cortical cells do not form narrow, ring-like preprophase bands (PPBs), which are plant-specific, cytoskeletal structures that predict the position of the division plane before mitosis. The results indicate that in the cortex but not in the epidermis, division plane positioning and patterning can proceed correctly in the absence of both a functional *TON1a* and PPB formation. Differences between tissues in how they respond to the signals that guide symmetric division orientation during patterning might provide the basis for organised organ growth in the absence of cell movements.

## Abstract (precis)

**Summary:** Adjacent tissues have different requirements for the precise positioning of the division plane, which is mediated at the intracellular level by the preprophase band that controls the pattern of cell division.

## INTRODUCTION

**Fig. 1.**
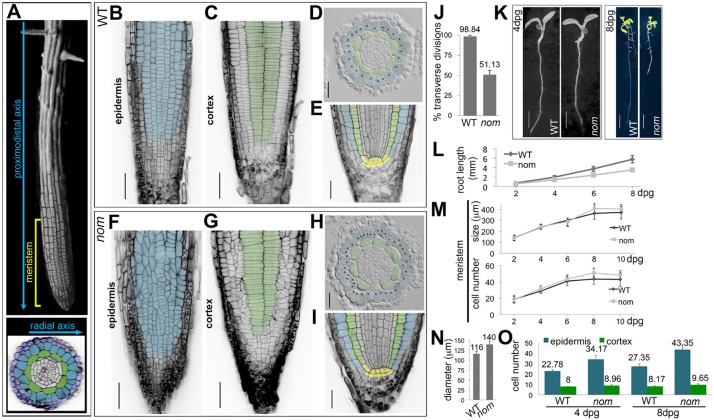
**The *nom* mutation affects the patterns of symmetric cell divisions in the *Arabidopsis* root meristem epidermis but not in the underlying cortical tissue.** (A) Top, SEM image of an *Arabidopsis* root with a superimposed confocal image of the root epidermis in the meristem; bottom, radial organisation of the root in a transverse section in the meristem: the epidermis (in blue) overlays the cortical tissue (in green) and on the outside is surrounded by the lateral root cap tissue (in dark blue). (B-I) Organisation of the root meristem in WT and *nom* mutant seedlings at 8 dpg. Note that in plants, the orientation of the cell walls, highlighted in black by propidium iodide (PI) staining, reflects the orientation of the division planes. Epidermal cells are pseudocoloured in blue, cortical cells in green. (B,C,F,G) Cellular organisation and division plane orientation in the epidermis and underlying cortical layer, images are single, longitudinal confocal sections of Schiff-PI stained meristems. Scale bars: 50 μm. (D,H) Transverse sections in the root meristem of resin-embedded seedlings; each black dot marks a single epidermal cell. Scale bars: 25 μm. (E,I) Single, median-longitudinal confocal sections in Schiff-PI-stained root meristems; stem cell niche are pseudocoloured in yellow. Scale bars: 25 μm. (J) percentage of transverse divisions in the root, meristematic epidermis of 8 dpg seedlings; WT *n*=19, *nom* *n*=20. (K) Whole seedlings at 4 and 8 dpg. Scale bars: 2 mm and 7.5 mm, respectively. (L-N) Data quantification. Root length (L) and meristem size (M) measured over time; for each time point, WT *n*=58, *nom* *n*=58 (L), WT *n*=40, *nom* *n*=40 (M); (N) diameter of the meristem; WT *n*=23, *nom* *n*=26; (O) number of epidermal and cortical cells scored in transverse sections within the root meristem at 4 and 8 dpg; at 4 dpg, WT *n*=319 epidermal and 113 cortical cells in 14 roots, *nom* *n*=786 epidermal and 206 cortical cells in 23 roots; at 8 dpg, WT *n*=629 epidermal and 188 cortical cells in 23 roots, *nom* *n*=1127 epidermal and 251 cortical cells in 26 roots. All data are means±s.d.; mean values are shown above bars for J,N,O.

In plants, where the presence of cell walls prevents cell movement, pattern formation studies can provide insights on how the control of cell division orientation leads to tissue and organ organisation. The *Arabidopsis* root is a simple system to investigate symmetric division control because perturbations in division patterns can be easily observed. In the root meristem, the zone where cells proliferate, the different cell types originate by asymmetric divisions from sets of stem cells. Subsequently, each cell population is expanded through regular, symmetric divisions, resulting in an organ composed of mono-layered tissues organised concentrically. During symmetric division in the epidermis and in the underlying cortical tissue, cells position their division plane in an anticlinal, transverse orientation and form a regular pattern of parallel files of cells arranged along the proximodistal axis of the root (Fig. 1A-C) (Dolan et al., 1993).

The preprophase band (PPB) is a transient array of microtubules that forms a narrow ring underneath the cell membrane during the G2 phase of the cell cycle and marks precisely the position of the division plane in the M phase. Mutant and drug studies suggest a crucial role for the PPB in the control of division plane orientation (Rasmussen et al., 2011, 2013). However, the few identified *Arabidopsis* mutants that are unable to form PPBs – the loss-of-function *tonneau1* (*ton1*) and *fass* (also known as *ton2*) – have impaired organisation of interphase microtubules, severe pleiotropic defects and extremely short roots where the regular division patterns are disrupted (Azimzadeh et al., 2008; Camilleri et al., 2002; Torres-Ruiz and Jurgens, 1994; Traas et al., 1995) and cannot provide information on cell division control at the tissue level. Here, we sought to identify the genetic basis of the pattern of epidermal and cortical symmetric divisions in the *Arabidopsis* root.

## RESULTS AND DISCUSSION

Through a forward genetic screen we isolated the recessive *nomad* (*nom*) mutant (see supplementary Materials and Methods). In the epidermis of WT roots, 98.84% of the symmetric divisions are in a transverse orientation relative to the proximodistal axis of the root, whereas in *nom*, only 51.13% are transverse and the other 48.87% are oblique (Fig. 1B,F,J). In the cortical tissue, cells divide again in a transverse orientation in the wild type (WT) and this is unchanged in *nom* (Fig. 1C,G). The concentric organisation of root tissues and the organisation of the stem cells niche reflect the ability of the stem cells to divide asymmetrically and to give rise to the different tissue types (Dolan et al., 1993), and they are the same in *nom* and WT (Fig. 1D,H,E,I). In *nom*, some defects in division orientation can been seen in the endodermis, the tissue subtending the cortex (Fig. 1D,H); however, division patterns along the root-hypocotyl axis during embryonic development are unaltered (Fig. S1). This indicates that the *nom* mutation alters the orientation of the symmetric divisions, but does not affect the root asymmetric divisions in the seedlings or the regular division patterns during embryogenesis.

Mutant *nom* seedlings can be discriminated from WT seedlings at 4 days post germination (dpg) by a small reduction in root length, which becomes more pronounced at 8 dpg (Fig. 1K,L), but along the proximodistal axis of the root, the meristem size of *nom* is unchanged compared with that of the WT (Fig. 1M). Within the radial dimension, 8 dpg *nom* root meristems were 20% wider than the WT (Fig. 1D,H,N). Although tissues were also mono-layered in the *nom* epidermis, they had 58.5% more cells than in the WT; by contrast, the increase in cell numbers was not as great in the *nom* cortex compared with the WT (+18%; Fig. 1,O). As division orientation determines to which growth axis of the organ the new cell will contribute, such a difference in epidermal cell number can be correlated with the oblique orientation of *nom* epidermal divisions.

**Fig. 2.**
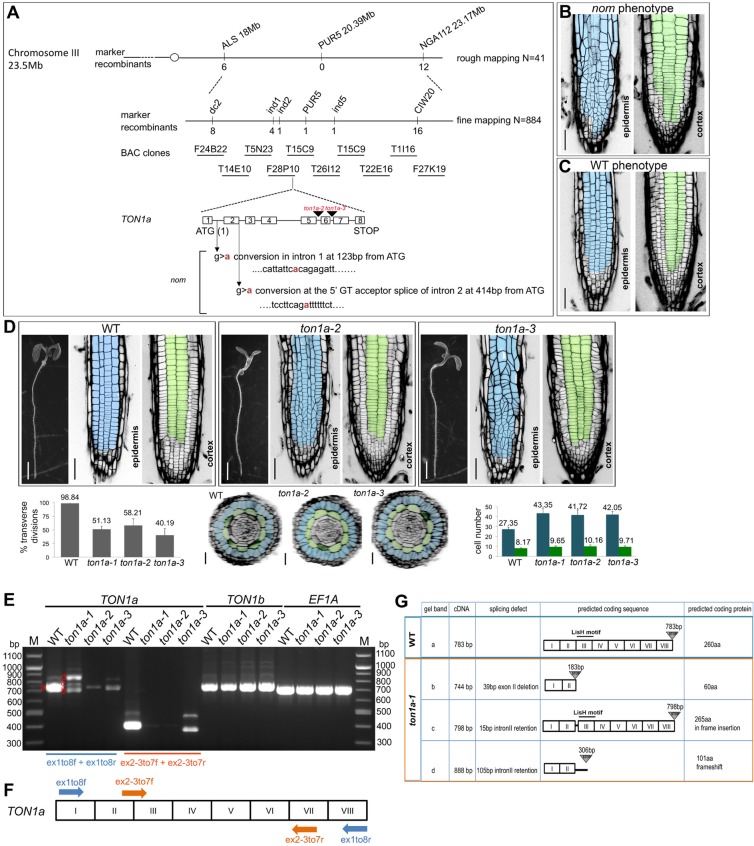
**The *nom* mutation maps to the *TON1a* gene and the three *ton1a* alleles identified are hypomorphic alleles of *TON1a*.** (A) Genetic mapping delimited the *nom* mutation to BAC clone F28P10. Schematic representation of the organisation of the gene, position of the point mutations relative to the start codon and position of the T-DNA insertions in the *ton1a-2* and *ton1a-3* alleles; *nom* was later renamed as the *ton1a-1* allele. (B,C) Confocal images of 4 dpg T2 seedlings segregating *nom* and WT phenotypes from T1 BASTA-resistant *nom* plants carrying the complementing construct. Epidermis is pseudocoloured in blue, cortex in green. Scale bars: 50 μm. (D) Phenotypic characterisation of the *ton1a-2* and *ton1a-3* alleles. Stereomicroscope images of whole seedlings and confocal images of the epidermis and cortex in the root meristem of WT and *ton1a-2* and *ton1a-3* alleles at 4 dpg, quantification of epidermal transverse divisions at 8 dpg; WT *n*=19, *ton1a-2* *n*=18, *ton1a-3* *n*=21; reconstructed transverse sections and respective quantification of epidermal and cortical cell numbers at 8 dpg, including those calculated for the *ton1a-1* allele; WT *n*=629 epidermal and 188 cortical cells in 23 roots, *nom* *n*=1127 epidermal and 251 cortical cells in 26 roots, *ton1a-2* *n*=751 epidermal and 183 cortical cells in 18 roots, *ton1a-3* *n*=883 epidermal and 204 cortical cells in 21 roots. Data are means±s.d. Scale bars: 2 mm for all seedlings, 50 μm for epidermis and cortex and 25 μm for transverse sections. (E-G) Molecular characterisation of the three *ton1a* alleles. (E) RT-PCR analysis with primer pairs specific for *TON1a* ex1to8f/r (in blue) produces in the *ton1a-1* allele three main transcripts (indicated with red b,c,d letters), and in the *ton1a-2* and *ton1a-3* alleles produces a severe reduction in the *TON1a* transcript compared with the WT. The transcripts in *ton1a-1* were cloned and sequenced to confirm they resulted from mis-splicing. Primer pairs ex2-3to7f/r (in orange) amplify only the correctly spliced WT transcript of *TON1a* and produce consistently barely detectable WT transcript for the *ton1a-1* allele. As a loading control, primer pairs that amplify the elongation factor 1A (*EF1A*) were used. (F) Schematic representation of primer locations on the *TON1a* cDNA; exons are indicated by roman numerals. (G) Schematic summary of RT-PCR and cloning results with the predicted amino acid sequences resulting from the mis-splicing of *TON1a* transcript in the *ton1a-1* allele and the location of the LisH dimerisation motif.

The *nom* mutation was mapped to the *TON1a* (At3g55000) locus (Fig. 2A). Complementation with a genomic fragment that restores the *nom* phenotype to WT (Fig. 2B,C) and the identification of two recessive, T-DNA insertion alleles, *ton1a-2* (GK-016D04) and *ton1a-3* (GK-727H06), which display epidermal-specific division defects like *nom*, confirmed its identity (Fig. 2D). The *nom* allele was renamed *ton1a-1. TON1a* lies in tandem to *TON1b*; they encode proteins that are 85% identical at the amino acid level and the two genes have been proposed to function redundantly (Azimzadeh et al., 2008). However, more recently, a unique function for the *TON1a* gene was hypothesised from biochemical studies (Spinner et al., 2013) and from a genetic interaction found between a *fass/ton2-15* allele and the *ton1a*-*te500* allele that has a WT root phenotype (Kirik et al., 2012). Our RT-PCR analysis shows that in the roots of the *ton1a-1*, *ton1a-2* and *ton1a-3* alleles there is a severe reduction in the *TON1a* transcript compared with that in WT roots and the *TON1b* gene is expressed as normal (Fig. 2E-G). This suggests that the consistent mutant phenotype we observed in the three *ton1a* alleles is caused by a reduction in the *TON1a* transcript and that the three *ton1a* alleles are hypomorphic alleles of *TON1a*. Thus, our data show the first direct, genetic evidence of a requirement for a functional *TON1a* gene alone in the control of symmetric division orientation within the root epidermis, but not in the underlying cortical tissue.

**Fig. 3.**
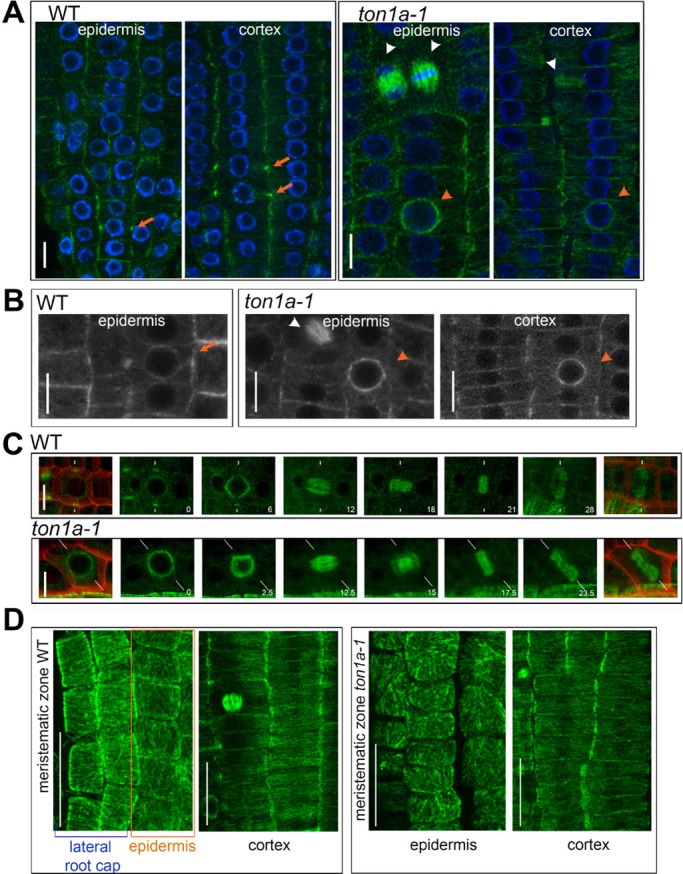
**In the root meristem, *TON1a* is required to form PPBs in both the epidermis and the cortex but does not control the organisation of interphase microtubules, which differs between the two tissues.** (A,B) Localisation of cytoskeletal structures in the meristematic epidermis of WT and *ton1a-1* roots, PPB (orange arrows), abundant microtubules surrounding the nucleus (orange arrowheads), spindle and cytokinetic phragmoplast (white arrowheads); all images are single, confocal sections. (A) Roots immunostained with α-tubulin (green), nuclei are stained blue with DAPI; WT *n*=6 roots observed 13 PPBs in the epidermis and 23 PPBs in the cortex, *ton1a-1* *n*=6 roots. (B) Representative close-up images of meristematic epidermal and cortical cells expressing *in vivo* the tubulin marker RFP-TUA5 (grey); WT *n*=12 roots observed 37 PPBs in the epidermis and 48 PPBs in the cortex, *ton1a-1* *n*=10 seedlings. (C) Stills from a confocal time-lapse movie of epidermal cells of WT and *ton1a-1* seedlings expressing GFP-β-tubulin from G2 phase to the end of M-phase (left to right); time is indicated in minutes, maximum intensity projections of confocal *z*-series, in the first and last stills the GFP images have been merged with PI images (red) to outline the cell walls, the white lines indicate the position of the division plane. (D) Representative images of maximum intensity projections of confocal optical sections in the epidermis and cortex of WT and *tona1-1* roots immunostained with α-tubulin (green); WT *n*=6, *ton1a-1* *n*=6. Note that in the first panels, epidermal cells (framed within an orange outline) are present in the same projections as lateral root cap cells. Scale bars: 10 μm (A-C) and 25 μm (D).

To test whether the root epidermal and cortical cells in *ton1a-1* form PPBs, we used anti-α-tubulin immunolocalisation. The narrow PPB ring of microtubules that forms at the cellular periphery can be seen as bright foci on each side of the cell in median confocal sections within WT cells (Fig. 3A). Instead, in median confocal sections within *ton1a-1* cells of the epidermal and cortical tissues, or of the inner tissues, we did not detect any bright foci, or structures resembling it and only detected more pronounced labelling around the nuclear periphery (Fig. 3A; Fig. S2), like in the studies of the loss-of-function *ton1* and *fass* mutants (Azimzadeh et al., 2008; Camilleri et al., 2002; Traas et al., 1995). Later division structures (spindle and cytokinetic phragmoplast) were normal in *ton1a-1* compared with the WT (Fig. 3A). Only occasionally were PPBs seen in lateral root cap cells, which surround the epidermis (data not shown). The absence of PPB formation in the epidermis and cortex was confirmed by examining *in vivo* the expression of the microtubule marker RFP-TUA5 in the *ton1a-1* mutant (Fig. 3B). Also, we were unable to detect PPBs in *ton1a-1* epidermal cells by *in vivo* time-course imaging using GFP-β-tubulin (Fig. 3C). Thus, *TON1a* appears consistently necessary to form the narrow PPBs in both the epidermis and the cortex, yet division orientation is correctly in place in the cortex, but not in the epidermis. Such ability of cells in the cortex to divide correctly is consistent with studies on cell cultures that have shown division plane can also be correctly placed without the formation of narrow PPBs (Chan et al., 2005; Marcus et al., 2005) and with the observations that not all plant species or all cell types form PPBs (Pickett-Heaps et al., 1999; Rasmussen et al., 2013). This suggests that PPB-independent mechanisms can guide division plane positioning in organised tissues.

The restriction of the defects in symmetric division orientation and patterning to the epidermal tissue in the three *ton1a* mutant alleles we have characterised raises the question of how such a tissue-specific phenotype might emerge. *TON1a* is part of the large TTP (TON1/TRM/PP2A) protein complex (Spinner et al., 2013). The possibility that mutations in *TON1a* affect the interactions between TON1a and the other components of the TTP complex in a tissue-specific manner seems unlikely because *ton1a* epidermal and cortical cells are equally unable to form narrow PPBs. An alternative possibility is that in the two tissues, cells have different requirements for a functional *TON1a* gene and for the formation of the PPB in the control of division plane orientation. We speculated whether the organisation of the interphase microtubules might underlie such different requirements. We found that, similar to what was also observed in WT meristems by Bichet et al. (2001), in both WT and *ton1a-1* meristems the interphase microtubules are not organised in epidermal cells and instead have a prevalent transverse organisation in cortical cells, which is parallel to the correct orientation of the division plane (Fig. 3D; Fig. S3). This suggests that in root meristematic cells, the *TON1A* gene is required for the formation of PPBs and not for the overall organisation of interphase microtubules. In addition, this result raises the possibility that the PPB might function to accurately fix division plane orientation in epidermal cells, where interphase microtubules and the cues responsible for division plane orientation are not aligned. Otherwise, where interphase microtubules are already aligned with such cues, PPB might be redundant, as seen in cortical cells. Such a possibility will need to be experimentally tested in further studies. Our results point to the existence of tissue-specific responses to the signals guiding the orientation of symmetric cell division during tissue patterning. This could be key to ensuring organised growth within proliferating meristems.

## MATERIALS AND METHODS

### Plant lines and growth conditions

The *Arabidopsis thaliana* T-DNA insertion lines GK-727H06 (N469786), GK-016D04 (N401480) and the *GFP-TUB6* line (N6550) were obtained from the Nottingham *Arabidopsis* stock centre (NASC). Descriptions of the following lines have been published: *GL2::GUS* (Masucci and Schiefelbein, 1996), GFP-MBD (Granger and Cyr, 2001) and RFP-TUA5 (Gutierrez et al., 2009). Phenotypic analysis was undertaken on *ton1a-1* mutant seedlings backcrossed three times into the parental *GL2::GUS* line. The *ton1a-1* phenotype was always compared against the phenotype of the parental line *GL2::GUS* (referred to here as WT). GK-727H06 (N469786) and GK-016D04 (N401480) T-DNA insertion lines were confirmed by PCR genotyping using primers listed in Table S1 as described in the supplementary Materials and Methods.

Seeds were surface sterilised in 10% sodium hypochlorite and sown on plates prepared with Murashige and Skoog (MS) salts (Duchefa), 1% sucrose and 0.5% phytagel (Sigma) medium (pH 5.8). Seeds were stratified in the dark at 4°C for 3 days and grown in a vertical position under continuous light at 28°C. The *nom* mutant was generated and characterised using standard techniques as detailed in the supplementary Materials and Methods. All experiments represent at least two independent replicates.

### Cell division orientation

In plants, cell wall orientation reflects cell division orientation and those cell walls oriented at a 90° angle with the proximodistal axis were scored as transverse divisions; cell walls whose orientation differed more than a 10° angle from the transverse orientation were scored as oblique. Using ImageJ (NIH), cell walls were scored within a rectangular frame of 100 μm (length)×50 μm (height) drawn over confocal *z*-series and centred at 100 μm from the quiescent centre of 8 dpg root tips stained with Schiff and propidium iodide (PI).

### Cell counting and meristem measurements

Epidermal and cortical cell numbers were counted on confocal, reconstructed transverse sections centred at 100 μm from the quiescent centre of 8 dpg root tips stained with Schiff-PI. The diameters of the meristems were measured on the same sections, excluding the lateral root cap tissue from the measurements. Root meristem size was measured on confocal, median, longitudinal sections of PI-stained roots using ImageJ software (NIH) as described previously (Dello Ioio et al., 2007).

### Immunocytochemistry

α-tubulin immunostaining of 4 dpg seedlings was carried out using established techniques (Collings and Wateneys, 2005; Sauer et al., 2006) with some modifications as in supplementary Materials and Methods.

### Microscopy and image processing

Confocal laser microscopy was performed with a Leica LCS SP5II microscope equipped with HyD detectors. The following wavelengths were used for fluorescence detection. Schiff-PI staining (Truernit et al., 2006): excitation, 488 nm and detection, 600-700 nm; GFP: excitation, 488 nm and detection, 493-550 nm; RFP: excitation, 561 nm and detection, 550-700 nm; DAPI: excitation, 405 nm and detection, 430-500 nm, as described in the supplementary Materials and Methods. Seedlings expressing the marker GFP-TUB6 were mounted in 50% MS liquid medium on slides and meristematic epidermal cells were imaged immediately.

Confocal images were processed with Image J64 software; reconstructed transverse sections were obtained by orthogonal projection of *z*-series collected at 0.4-0.5 μm intervals. Maximum intensity projections were done on *z*-series collected at 0.5 μm intervals for GFP-TUB6. Photoshop CS6 was used to prepare and pseudocolour the images for the figures. Images of longitudinal confocal sections and reconstructed transverse sections of PI-stained seedlings illustrating the organisation of tissues were inverted and their levels adjusted so that the PI staining, which outlines the profile of the cells, is in black.
